# Source-renewing ectopic ApoB-TRL lipolysis in non-resolving ARDS: a testable spatial hypothesis

**DOI:** 10.3389/fimmu.2026.1859209

**Published:** 2026-08-31

**Authors:** Yuwu Liu, Ping Shi

**Affiliations:** 1 Department of Orthopedics, Guangyuan Central Hospital, Guangyuan, China; 2 Department of Respiratory Medicine, Guangyuan Central Hospital, Guangyuan, China

**Keywords:** acute respiratory distress syndrome, cytokines, lipoprotein lipase, macrophage efferocytosis, pulmonary microcirculation, spatial propagation, tissue hypoxia, triglyceride-rich lipoproteins

## Abstract

Acute respiratory distress syndrome (ARDS) may remain regionally permeable and poorly reparative after the initiating insult has weakened. We propose that, in a biologically enriched subset of non-resolving ARDS, repeated entry of circulating apolipoprotein B-containing triglyceride-rich lipoproteins (ApoB-TRLs) into an injured perivascular or interstitial compartment provides a renewable substrate for local injury. Catalytically active lipoprotein lipase (LPL) converts particle triglyceride (TG) to free fatty acids (FFAs). When FFA generation exceeds albumin buffering, cellular disposal, and lymphatic removal, redox and lipid-mediator signaling induces a focused cytokine program and recruits neutrophils. Oxidants, proteases, and neutrophil extracellular trap (NET)-fibrin complexes then injure the glycocalyx, junctions, matrix, and epithelium, permitting entry of the next particle cohort. Renewed source-resolved input, rather than lipid abundance alone, distinguishes a self-maintaining loop from a finite amplifier. Effective LPL activity, a macrophage brake, regional hypoxia, and slow structural locks modify circuit phase and reversibility but do not define loop closure. Spatial progression is predicted to be signal-first: mobile inflammatory or coagulation relays prime a remote endothelial gate, while fresh substrate is supplied by the recipient microcirculation. The decisive prediction is ordered recurrence in the same microdomain: source-resolved entry and conversion precede inflammatory execution and barrier loss, and interrupting a verified downstream executor after matching the first input prevents a second particle input. Failure of this sequence would narrow or reject the hypothesis.

## 1 Introduction

ARDS is not a single biological trajectory. Permeability edema, endothelial and epithelial injury, impaired alveolar fluid clearance, inflammatory-cell recruitment, and immunothrombosis explain much of its early pathophysiology, yet patients and lung regions differ markedly in persistence and repair (1–5). Spatial and single-cell studies further show that destructive and reparative programs can coexist in anatomically distinct microenvironments (6–8). A progression hypothesis must therefore explain not only how an established focus persists after the initiating trigger has attenuated, but also how a new focus becomes established elsewhere.

We propose a bounded mechanism for a subset of non-resolving ARDS. Pulmonary microvascular injury permits circulating ApoB-TRLs to enter a perivascular or interstitial compartment in which active LPL is present. Local extracellular TG-to-FFA conversion generates inflammatory exposure; a cytokine-neutrophil-NET execution layer converts that exposure into structural barrier injury; and the deteriorated barrier admits another particle cohort. The critical claim is source renewal. Without renewed particle input, ectopic lipolysis is a finite amplifier. With renewed input, lipid-cytokine-barrier coupling regenerates its own substrate and becomes a progression engine.

The proposal has four linked parts: spatial lipid mislocalization creates the reaction compartment; conversion and cytokine-mediated structural execution close the local loop; macrophage function, effective LPL activity, hypoxia, and accumulated structural damage determine whether the loop resolves or locks in; and mobile signals establish distant permissive gates before locally supplied ApoB-TRLs seed a new loop. The hypothesis does not claim that all ARDS is lipid-driven, that plasma TG identifies the endotype, or that any single lipid-lowering, anti-inflammatory, or oxygen strategy is clinically indicated. Its purpose is to specify the temporal order and perturbation results that would support or reject a source-renewing mechanism.

## 2 Spatial mislocalization creates a source-resolved reaction compartment

The alveolar-capillary barrier contains two serial boundaries. The endothelial glycocalyx and intercellular junctions separate plasma from the basement-membrane-pericyte-extracellular-matrix unit, while the alveolar epithelium separates that abluminal space from the airspace. ARDS can injure both boundaries, but transport of small solutes, albumin, and lipoprotein particles need not increase in parallel (9–13). The proposed initial reaction compartment is therefore perivascular or interstitial. ApoB or TG detected in edema fluid or bronchoalveolar lavage fluid (BALF) may reflect later epithelial passage and cannot by itself identify where the reaction began.

The candidate renewable substrate is TG carried predominantly in very-low-density lipoproteins and remnant particles. Chylomicrons may contribute during enteral feeding or postprandial sampling but should not be assumed in fasting critically ill patients. TG is carried within the particle core rather than covalently bound to ApoB; consequently, source attribution requires ApoB-48 or ApoB-100, particle size or density, and core TG species to be measured together. Sheep lung-lymph studies demonstrate that plasma lipoproteins can enter pulmonary interstitial lymph and undergo remodeling, establishing anatomical plausibility rather than pathological influx in human ARDS (14–16). Edema-fluid and BALF lipidomics demonstrate lipid heterogeneity but do not reliably separate plasma particles from surfactant, cell membranes, or extracellular vesicles (17–19).

LPL is the candidate converter. Under physiological conditions, LPL is synthesized on the abluminal side and transported by glycosylphosphatidylinositol-anchored high-density lipoprotein-binding protein 1 (GPIHBP1) to the luminal endothelial surface, where the GPIHBP1-LPL complex supports TRL margination and hydrolysis (20, 21). Extraluminal LPL is therefore not intrinsically pathological. The proposed lesion is sustained colocalization of escaped ApoB-TRLs with catalytically active LPL outside the lumen because of barrier failure, matrix retention, disturbed trafficking, or production by recruited monocytes or macrophages. Human monocytes and cultured alveolar macrophages can secrete active LPL, although this does not establish a pathogenic interstitial source in ARDS (22).

Historical lung experiments provide proof of possibility. In an isolated perfused canine pulmonary lobe, heparin-releasable pulmonary LPL converted circulating TG to FFA in association with edema, shunt, and reduced compliance; experimental studies identified pulmonary macrophages as an LPL source; and LPL-dependent FFA generation was detected in bronchoalveolar lining fluid (23–25). These observations show that pulmonary TG hydrolysis can occur. They do not prove extravascular ApoB-TRL entry, temporal precedence, or recurrent barrier-dependent input in human ARDS.

Accordingly, an ectopic reaction compartment requires three simultaneous findings: source-resolved ApoB-TRLs beyond the lumen, catalytically active extracellular TG-lipolytic enzyme, and measurable local TG-to-FFA conversion. Particle localization without conversion indicates leak; LPL protein without activity is non-diagnostic; and FFA without source linkage may derive from plasma, membranes, or surfactant. Surfactant degradation should instead be identified by phosphatidylcholine (PC) loss, particularly dipalmitoylphosphatidylcholine (DPPC), an increased lysophosphatidylcholine-to-phosphatidylcholine (LPC: PC) ratio, secretory phospholipase A2 (sPLA_2_) activity, impaired surfactant proteins or surface activity, and absence of particle-resolved ApoB linkage (26–29). LPL hydrolyzes the neutral TG core of ApoB-TRLs, whereas sPLA_2_ acts on surfactant phospholipids and generates lysophospholipids and fatty acids; intracellular neutral-lipid hydrolysis after macrophage uptake is a separate cellular process. **Table 1** defines the minimum evidence needed to separate these processes.

**Table 1:** Distinct lipid processes and the evidence required to separate them.

Lipid process	Minimum evidence	Interpretive boundary
Circulating ApoB-TRL entry	ApoB-48/ApoB-100, particle size or density, plasma-matched core TG species, correction for nonspecific leak	Entry is established; local hydrolysis is not
Extracellular TG conversion	Active LPL, isotope-linked TG-to-FFA/glycerol/partial glycerides, spatial colocalization	Required reaction step; LPL abundance alone is insufficient
Albumin-bound plasma FFA	FFA species, unbound FFA or FFA:albumin ratio, paired plasma-to-lung gradient	Parallel circulating input; do not attribute automatically to TRL hydrolysis
Injured-cell membrane lipids	Cell-death markers, membrane phospholipid species, cell-of-origin markers, spatial relation to necrosis	Usually downstream injury; not evidence of ApoB-TRL entry or extracellular TG conversion
Extracellular-vesicle-associated lipids	Vesicle markers, cell-of-origin markers, vesicle-resolved lipidomics, vesicle/non-vesicle fractionation	Intercellular signaling cargo; separate from particle escape and injured-cell membrane release
Intracellular lipid droplets	Cell-resolved neutral-lipid imaging, perilipin proteins, and uptake or turnover tracers	May indicate buffering, overload, or failed disposal; not direct evidence of extracellular lipolysis
Surfactant degradation	DPPC/PC loss, LPC: PC ratio, sPLA_2_ activity, surfactant proteins and surface activity	Phospholipid degradation; not evidence of ApoB-TRL TG conversion
Oxidized lipids and eicosanoids	Molecular species, oxidation or enzymatic pathway, cell localization, time course	Mediator or response; role depends on source and temporal position

## 3 The source-renewing lipid-cytokine-barrier engine

The biologically relevant exposure is flux rather than bulk lipid abundance. Let J_TRL,i_ denote particle-TG influx into lung region i, P_TRL,i_ particle-specific permeability, and C_TRL,p_ the paired circulating particle-TG concentration. The minimal input relation is:


$${\text{J}}_{\text{TRL,i}}\left(\text{t}\right)\;=\text{ f}[{\text{P}}_{\text{TRL,i}}\left(\text{t}\right),{\text{ C}}_{\text{TRL,p}}\left(\text{t}\right)],\;\partial \text{ J}/\partial \text{ P }>\;0,\;\partial \text{ J}/\partial \text{ C}>0$$

Let T_i_ denote extravascular particle TG, F_i_ bioavailable FFA, E_i_ effective extracellular LPL activity, v_LPL_ local hydrolytic flux, J_FFA,i_ independently measured plasma-FFA input, and k_T_ and k_F_ net removal. Qualitative mass balances are:


$${\text{dT}}_{\text{i}}/\text{dt}={\text{J}}_{\text{TRL,i}}\left(\text{t}\right)-{\text{v}}_{\text{LPL}}[{\text{T}}_{\text{i}}\left(\text{t}\right),{\text{ E}}_{\text{i}}\left(\text{t}\right)]-{\text{k}}_{\text{T}}{\text{T}}_{\text{i}}\left(\text{t}\right)$$

$${\text{dF}}_{\text{i}}/\text{dt}={\text{y v}}_{\text{LPL}}[{\text{T}}_{\text{i}}\left(\text{t}\right),{\text{ E}}_{\text{i}}\left(\text{t}\right)]\;+{\text{ J}}_{\text{FFA,i}}\left(\text{t}\right)\;-{\text{ k}}_{\text{F}}{\text{F}}_{\text{i}}\left(\text{t}\right)$$

These relations are not a fitted quantitative model. Albumin binding is a reversible buffer that changes unbound or membrane-proximal FFA activity and should be measured separately; k_F_ represents net irreversible cellular metabolism, export, or lymphatic removal. After a finite particle pulse, TG may decline while FFA rises. During continuing leak, J_TRL_ can replenish TG faster than hydrolysis and removal, allowing both pools to rise. Cross-sectional TG-FFA correlation is therefore not a decisive test. Source-resolved flux, time-lagged sampling, and paired plasma measurements are required.

When local FFA generation exceeds buffering and disposal, saturated long-chain fatty acids and oxidized lipid species can alter membrane organization, amplify Toll-like-receptor-associated priming, promote endoplasmic-reticulum stress and ceramide generation, and impair mitochondrial function. These routes converge on NADPH-oxidase- and mitochondria-derived reactive oxygen species, nuclear factor-kappa B, mitogen-activated protein kinases, and inflammasome-associated signaling (30–36). Palmitate should not be treated as a universal direct Toll-like receptor 4 ligand, and the NADPH oxidase family should be regarded as a convergence node rather than a lipid receptor.

The predicted output is a spatially focused cytokine program, not a non-specific cytokine storm. TNF-α and IL-1β can destabilize endothelial and epithelial junctions and increase leukocyte adhesion; in human lung endothelial cells, TNF-α induces VE-cadherin phosphorylation and increases paracellular albumin flux (37). IL-6 can sustain endothelial and acute-phase activation, CXCL8/IL-8 recruits and prolongs neutrophil survival, and CCL2 expands monocyte input. Persistent BALF cytokine elevation is associated with poor ARDS outcome, but systemic or airspace abundance does not prove that ectopic lipolysis initiated the signal (3, 4, 38). The model therefore prespecifies a compact local module - TNF-α, IL-1β, IL-6, CXCL8, and CCL2 - and predicts that its rise follows source-linked FFA-redox flux but precedes structural deterioration and renewed particle entry.

Lipid mediators can sharpen the same execution program. Leukotriene B4 provides a neutrophil relay, while prostanoid balance modifies endothelial and epithelial barrier signaling, platelet-vascular behavior, and regional perfusion. Thromboxane A2 favors vasoconstriction and platelet activation, whereas prostacyclin opposes platelet activation and supports vasodilation (39–42). However, lipid-mediator changes alone do not establish ApoB-TRL entry.

Neutrophils provide the rapid structural execution layer. Cytokines and lipid mediators recruit and prime them; NADPH oxidase 2 (NOX2), myeloperoxidase, elastase, matrix metalloproteinases, and NETs then injure the glycocalyx, endothelial junctions, basement-membrane-pericyte-matrix unit, surfactant interface, and alveolar epithelium. NET chromatin carries histones, proteases, oxidized DNA, and associated enzymes and binds platelets, complement, tissue factor, and fibrin. The resulting scaffold promotes endothelial injury, microvascular obstruction, thrombosis, and delayed immune resolution (43–46). NET identification requires orthogonal molecular markers plus imaging rather than cell-free DNA alone, and NET or peptidylarginine deiminase 4 (PAD4) interruption cannot be assumed to be uniformly beneficial (47).

**Figure 1:**
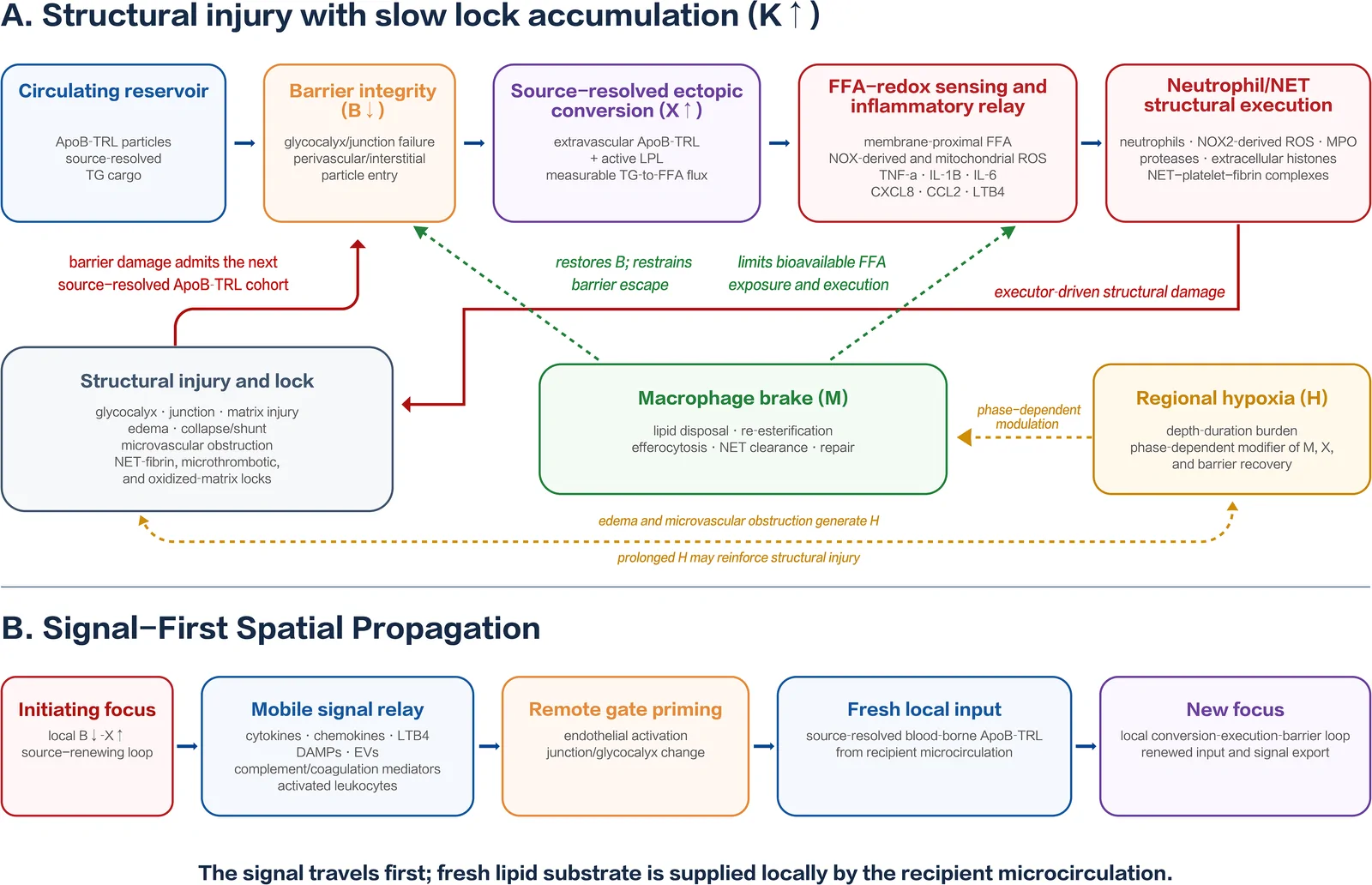
Source-renewing ectopic ApoB-TRL lipolysis and signal-first spatial propagation. **(A)** Barrier failure permits circulating ApoB-containing triglyceride-rich lipoproteins (ApoB-TRLs) to enter a perivascular or interstitial compartment containing active lipoprotein lipase (LPL). Extracellular triglyceride (TG)-to-free fatty acid (FFA) conversion activates FFA-redox sensing, cytokine and lipid-mediator relay, and subsequent neutrophil/NET structural execution. Injury to the glycocalyx, endothelial junctions, matrix, and microvasculature permits renewed ApoB-TRL entry and closes the loop. The macrophage brake limits FFA exposure and supports clearance and repair, whereas regional hypoxia acts as a phase-dependent modifier. **(B)** Mobile inflammatory and coagulation signals prime a remote endothelial gate before fresh ApoB-TRL substrate enters from the recipient microcirculation and establishes a new local focus. Thus, the signal travels, whereas the lipid substrate is supplied anew locally.

The loop is closed only when this downstream execution creates the next upstream input. After the first particle cohort and initial barrier insult have been matched, selective interruption of a cytokine, redox, or NET-associated executor should prevent or reduce entry of a second source-resolved ApoB-TRL cohort. Persistence of cytokines or tissue injury without renewed input defines a finite amplifier, not a source-renewing engine. This causal sequence is summarized in **Figure 1A**.

## 4 Brakes, hypoxic regulation, and phase transitions

The core engine does not determine fate by itself. Perivascular/interstitial macrophages and recruited monocytes occupy the reaction space; resident alveolar macrophages mainly survey the airspace. These populations link lipid handling to debris clearance and repair. ARDS impairs alveolar-macrophage efferocytosis and NET clearance, while defined recruited macrophage states can support alveolar regeneration (48–53). Cell abundance or a single polarization marker cannot establish whether this protective function is intact.

We define the macrophage brake, M, as a compartment-specific capacity with three prespecified domains: lipid disposal; efferocytosis and NET clearance; and endothelial or epithelial repair support. The domains should be reported separately as well as integrated. Sufficient M can prevent ectopic conversion from recruiting a self-renewing execution layer; progressive failure predicts persistent conversion, unresolved debris, and failure to restore the barrier. This functional definition avoids treating activated macrophages as uniformly protective or injurious.

Effective LPL activity is phase-dependent. High extracellular hydrolytic flux can increase local FFA exposure, whereas very low activity may reduce the FFA arm while allowing interstitial particle TG to accumulate. The outcome depends on influx, buffering, disposal, and whether retained TG accompanies energetic or necrotic injury. Direct activity and isotope-linked flux, rather than LPL abundance, are required to identify the phase.

Regional hypoxia arises through permeability edema, diffusion limitation, collapse and shunt, NET-fibrin or platelet-rich obstruction, inflammatory oxygen demand, and mitochondrial inefficiency. H denotes cumulative local depth-duration burden rather than arterial oxygenation. Modest oxygen limitation can support macrophage survival, epithelial repair, and metabolic adaptation for efferocytosis (54–56), whereas prolonged hypoxia may impair macrophage lysosomal activity (57). Evidence that acute hypoxia reduces LPL activity comes from human adipose tissue, and VEGF-A/VEGFR2-Y949 permeability evidence comes from chronic hypoxic pulmonary hypertension; neither directly establishes an acute ARDS particle-permeability switch (58, 59).

The prediction is biphasic and component-specific: increasing H may initially preserve adaptation and later reduce M, alter effective LPL flux, or increase particle permeability. Hypoxia is not required for the first loop closure. If matched structural injury fully explains renewed entry after H is manipulated, hypoxia should be classified as a marker or modifier rather than the source-renewing gate. Clinical oxygen-target trials should not be conflated with these local mechanistic tests (60).

K represents organized NET-fibrin, persistent microthrombi, oxidized or cross-linked matrix, epithelial denudation, and maladaptive repair. Accumulating K reduces reversibility even after substrate input or cytokine signaling falls. M, effective LPL activity, H, and K regulate the B-X engine; none replaces source renewal as its defining criterion.

## 5 Signal-first spatial propagation

A closed local circuit explains persistence but not spatial progression. Soluble cytokines and chemokines, leukotriene B4, damage-associated molecular patterns, extracellular vesicles, complement-coagulation mediators, activated platelets, and mobile leukocytes can leave an established focus and influence a distant vascular bed (38–40, 44, 45, 61–63). The ApoB-TRL substrate need not travel from the initiating lesion. It is already available in the recipient circulation and enters only when the recipient endothelial gate becomes sufficiently permissive.

The predicted sequence at an advancing edge is therefore signal-first and substrate-second. A mobile relay should first produce endothelial activation, glycocalyx or junctional change, leukocyte adhesion, or a measurable increase in particle-specific permeability. A distinguishably labeled cohort of fresh circulating ApoB-TRLs should then enter the recipient region, encounter local active LPL, and generate source-linked FFA. The recipient focus becomes persistent only if its own conversion-execution-barrier sequence creates a further local particle input. Thus, propagation transfers permissive information, whereas each focus obtains its lipid substrate locally.

A two-region experiment can distinguish this topology from lipid transport. The initiating region and a spatially separated recipient region should be sampled at short intervals while plasma particles receive temporally distinguishable labels. Remote gate activation preceding recipient-region particle conversion would support signal-first propagation. Detection of initiating-focus lipid products in the recipient region before gate activation, failure of the recipient region to acquire local TG-to-FFA conversion, or propagation despite blockade of all measurable mobile relays would narrow or reject the proposed topology. **Figure 1B** illustrates this sequence.

## 6 Decisive tests and regional state integration

For research use, non-resolution should be defined prospectively as persistent physiological non-improvement with permeability, injury, or failed-repair signals over 48–72 hours after stabilization; this is not a clinical diagnosis. Serial plasma and lung-compartment samples should be interpreted with nutrition, ventilation, perfusion, extracorporeal support, drug exposure, and severity measures. Urea or total-protein normalization alone cannot correct dilution, epithelial transfer, blood contamination, and particle-specific transport (64–66).

Source and reaction must be resolved in the same specimen. ApoB-48/ApoB-100, particle size or density, core TG species, active LPL, partial glycerides, glycerol, FFA species, unbound FFA or the FFA-to-albumin ratio, and isotope linkage should be measured together. Paired plasma measurements distinguish increased tissue entry from increased circulating substrate. In a dual-pulse design, the first labeled cohort establishes entry and conversion, whereas a distinguishable second cohort tests renewed input.

Spatial transcriptomics, proteomics, imaging mass spectrometry, multiplex histology, and particle-resolved imaging should test whether entry and conversion microdomains precede cytokine-active macrophage or endothelial states, neutrophil-NET neighborhoods, hypoxic burden, and repair failure. Existing spatial studies identify anatomically distinct macrophage, endothelial-injury, epithelial-transition, fibroblast, neutrophil, and repair niches in severe lung injury, but none simultaneously resolves ApoB-TRL entry, active LPL, or TG-to-FFA flux; they therefore support the spatial premise rather than the complete lipid mechanism (6–8, 67). H should not be inferred from PaO_2_ alone; it should integrate local oxygen or validated hypoxia labeling with perfusion, microthrombotic context, lactate-redox indices, and spatial hypoxia-response modules. Concordance across modalities is more credible than any single marker.

Causal closure requires matched initial conditions and verified target engagement. After the first barrier insult and labeled particle input are matched, experiments should first test extracellular TG conversion, the prespecified cytokine-redox-NET execution layer, and macrophage disposal-repair capacity in separate, target-engaged arms. In a lock-in phase, a synchronized factorial arm should combine partial control of particle entry or ectopic LPL flux with transient suppression of cytokine-NET execution and restoration of macrophage disposal-repair capacity; verified single-node arms provide the comparators. Every arm should use the same causal endpoint: entry and conversion of the second particle cohort, rather than only oxygenation, inflammatory markers, or histological injury. A regional hypoxia perturbation should span adaptive and deeper or longer exposure without causing nonspecific lethal injury. If reducing active LPL lowers FFA injury but increases retained TG, that is a predicted phase shift rather than simple model failure.

For longitudinal integration, each lung region i is represented by a five-variable phenomenological state:


$${\text{S}}_{\text{i}}\left(\text{t}\right)=[{\text{B}}_{\text{i}}\left(\text{t}\right),{\text{ X}}_{\text{i}}\left(\text{t}\right),{\text{ H}}_{\text{i}}\left(\text{t}\right),{\text{ M}}_{\text{i}}\left(\text{t}\right),{\text{ K}}_{\text{i}}\left(\text{t}\right)]$$

B_i_ denotes alveolar-capillary barrier integrity; X_i_, source-resolved ectopic ApoB-TRL TG-to-FFA flux; H_i_, cumulative regional hypoxic burden; M_i_, macrophage lipid disposal, efferocytosis/NET clearance, and repair capacity; and K_i_, slow-relaxing structural locks. B and X define the source-renewing circuit, H and M regulate gain and recovery, and K captures delayed loss of reversibility. The vector is neither a clinical score nor an identified quantitative system; it organizes region-matched longitudinal sampling and intervention timing.

**Table 2** lists supporting and rejecting outcomes. The strongest validation is an ordered sequence in the same microdomain followed by selective prevention of the second input through a target-engaged downstream intervention. A lipid-linked endotype should be claimed only if this signature adds information beyond plasma lipids, established ARDS subphenotypes, ventilation, perfusion, nutrition, and conventional inflammatory or endothelial markers.

**Table 2:** Prespecified predictions and falsification rules.

Prediction	Supporting result	Result that narrows or rejects the claim
Source-resolved entry and conversion	Extravascular ApoB-TRLs precede isotope-linked local TG-to-FFA flux	No entry rejects the substrate; entry without conversion indicates leak
Cytokine execution	FFA-redox flux precedes the prespecified local cytokine module; blockade reduces structural execution	Cytokines appear only after injury, or blockade changes markers without changing injury
Local feedback closure	Downstream interruption prevents entry/conversion of the second cohort after the first is matched	Persistence without renewed input indicates a finite amplifier
Macrophage brake	Compartment-specific rescue improves lipid disposal, clearance, and barrier recovery with target engagement	Target engagement without improvement narrows the model to an M-independent phase
Hypoxic regulation	Graded depth-duration H produces an adaptive range followed by component-specific loss of M, altered X, or increased permeability	No phase change narrows the biphasic claim; H-independent renewal rejects H as a decisive gate
Signal-first propagation	Remote gate activation precedes fresh local ApoB-TRL entry, conversion, and local recurrence	Initiating-focus lipid arrives first, or recipient regions never acquire local conversion
Synchronized multi-node design	After lock-in, a phase-matched combination targeting renewed input or conversion, inflammatory execution, and repair failure outperforms target-engaged single-node arms for preventing second-cohort entry or conversion	No added benefit supports a simpler dominant-node model

## 7 Discussion and conclusion

The proposed framework assigns distinct roles to location, flux, execution, regulation, and propagation. Spatial mislocalization places a renewable plasma-derived substrate in an ectopic reaction compartment. Extracellular TG-to-FFA conversion generates a local exposure; a focused cytokine and lipid-mediator program recruits neutrophils; and redox, protease, and NET-fibrin execution converts biochemical exposure into structural barrier injury. Renewed entry of source-resolved ApoB-TRLs distinguishes a self-maintaining progression engine from a finite inflammatory pulse.

The central uncertainty is the absence of direct evidence for this complete ordered sequence in human ARDS. TRL subclasses may differ in entry; colocalized LPL may be inactive; protein-rich edema may buffer FFA; cytokines may arise independently from infection, sepsis, or ventilation; macrophage LPL may be protective in some phases; and surfactant degradation, cell death, nutrition, or extracorporeal support may reproduce parts of the signature. TNF-α-associated albumin permeability does not establish ApoB-particle permeability, hypoxia-sensitive LPL evidence comes from adipose tissue, and VEGFR2-Y949 permeability evidence comes from chronic hypoxic pulmonary hypertension rather than acute ARDS (37, 58, 59). These alternatives make isotope-resolved flux, particle-specific permeability, within-region temporal order, and intervention target engagement indispensable.

The framework nevertheless generates stage-dependent predictions. Before renewed input is established, barrier recovery or reduction of excessive ectopic conversion may be sufficient to terminate the process. After cytokine-neutrophil-NET execution is active, interrupting only the initiating lipid node may leave structural injury self-sustaining. Loss of M or accumulation of K may further reduce reversibility. Conversely, when effective LPL activity has already fallen, additional LPL suppression could reduce FFA generation while worsening particle-TG retention. These predictions justify state-timed experiments, not clinical treatment recommendations.

The hypothesis should be rejected or narrowed if source-resolved particles do not enter the proposed compartment, if they enter but are not locally converted, if conversion follows rather than precedes structural destruction, if downstream interruption does not alter the second particle input, or if distant foci arise without prior gate activation and local substrate conversion. Until those tests are completed, ectopic ApoB-TRL lipolysis remains a falsifiable explanation for a subset of non-resolving ARDS. Its value is to specify where the lipid originates, how cytokine and neutrophil execution renews its source, which mechanisms brake or lock the circuit, and how a mobile signal can establish a new focus without requiring lipid to travel from the initiating lesion.

## Data Availability

The original contributions presented in the study are included in the article. Further inquiries can be directed to the corresponding author.
